# An experimental evaluation of vibration patterns arising from vortex flow in mixed flow pumps a case study in Upper Egypt

**DOI:** 10.1038/s41598-025-25110-4

**Published:** 2025-11-18

**Authors:** Dalia M. S. El-Gazzar, Abdallah H. I. Abo-Elnil, Mostafa E. A. Elsayed

**Affiliations:** 1 Mechanical Engineering Department, Mechanical and Electrical Research Institute, National Water Research Center, Cairo, Egypt; 2 Mechanical Engineering Department, Faculty of Engineering and Technology, Badr University in Cairo (BUC), Cairo, Egypt; 3 Mechanical Engineering Department, Faculty of Engineering at Shoubra , Benha University, Cairo, Egypt

**Keywords:** Mixed pump, Vibration analysis, Vortex, CFD, Signal recognition, Electrical and electronic engineering, Mechanical engineering, Computational science

## Abstract

Mixed flow pumps, characterized by high flow rate and moderate head, are the preferred choice for pumping station installations. Nevertheless, the development of inlet vortices significantly impairs the performance of mixed-flow pumps, threatening operational stability and safety. This focuses on a large pumping station where unstable and fluctuating vibration levels were observed across four pumping units during extended operation. Over a continuous 6 h period, total vibration velocity levels were recorded at 2 h intervals. Both velocity and acceleration spectra were analyzed at all measurement sites to investigate the pronounced fluctuations in vibration. Computational fluid dynamics (CFD) simulations were employed as the primary method to realistically visualize the complex turbulent flows occurring in the pump intake under various operating conditions. The results showed that vibration magnitudes increased notably near the blade-passing frequency whenever a vortex was present. Furthermore, a vortex’s presence was successfully recognized at measurement points due to sudden spikes in vibration intensity. The formation, pattern, and regularity of vortex flows were examined using numerical simulation to address this issue. Based on the numerical findings, recommendations for improving and modifying the pumping station inlet design were proposed. Experimental validation confirmed that constructing a curtain wall to reduce free vortices above and below the water surface is an effective and practical solution to mitigate vortex formation. Maximum vibration level reduced by about 73% after execution of the curtain wall to the pump intake. This research presents an innovative and effective solution to an actual problem occurring in a large pumping station. This solution is considered a scalable procedure which can be applied to any pumping station exposed to the problem of vortices.

## Introduction

In the context of fluid transfer, the selection between axial flow pumps and mixed flow pumps is determined by the specific hydraulic requirements of the application, primarily the desired head and flow rate. Axial flow pumps are best suited for applications demanding high flow rates with a low head, as their design propels fluid parallel to the pump axis, maximizing volume transfer with minimal pressure increase. Conversely, mixed flow pumps represent a versatile hybrid, providing balanced performance of both moderate head and high flow. This makes them the optimal choice for tasks requiring a significant increase in pressure in addition to substantial fluid volume^1,2^.

Several studies have addressed related phenomena: Fan Yang et al.^3^, examined the hydraulic stability and internal flow characteristics of a vertical submersible pump stations in both forward and reverse operating modes. Zhang et al.^4^. explored the generation and evolution of tip leakage vortices in axial flow pumps, assessing the effects of gap width and the cavitation on vortex formation through experimental and numerical methods. Chen et al.^5^ utilized hybrid numerical techniques to study the structural vibration and noise generated by flow within axial-flow pumps, validating pressure fluctuations experimentally and identifying the blade-passing frequency as the dominant vibration frequency.

Vortex formation at pipe intakes is a critical concern affecting pumping station performance. Sabouki et al.^6^. combined experimental and numerical approaches study vortex generation in water intake systems. Xiaohui Wang et al.^7^ used detached eddy simulation to analyze vortex evolution, demonstrating that vortices consistently form at the impeller inlet region and maintain stable size and location throughout operation.

Jie Gong et al.^8^ investigated the impact of cavitation and vortex dynamics on axial flow pump performance, showing that cavity growth at lower ambient pressures induces earlier vortex merging. Wang et al.^9^ examined the long-term effects of cavitation-induced vibrations, highlighting the increase in frequency amplitude with rising flow rates and the non-stationary behavior of vibration signals.

A substantial body of work further explores the impact of vortices on pump performance through both numerical and experimental methods^10,11^. These studies reveal the ability to detect vortex-induced vibrations in real-time and inform open-design vortex pump impeller development^12,13^.

Ramadhan Al-Obaidi ^14,15^ applied CFD methodologies for qualitative and quantitative flow field analyses in mixed flow pumps, incorporating frequency domain pressure variation analysis under different operating conditions. Additionally,

investigations into hydrodynamic and vibratory characteristics of mixed flow pump system highlight strong correlations between vibration deformation, hydraulic forces, and impeller pressure distribution^16–19^.

CFD studies utilizing the standard k-ε turbulence model and the ANSYS Fluent software provide insights into complex centrifugal and mixed-flow pump patterns, emphasizing the influence of impeller blade geometry on efficiency and noise production^20,21^.

Fluid-induced instabilities, often manifesting as high amplitude sub-synchronous vibrations caused by vortices or whirl phenomenon, typically occur when pumps operate below their optimal efficiency point. These instabilities arise where shear layers exist between fundamental and circulating flows. Understanding these mechanisms aids in improving pump-turbine reliability and operational efficiency^22–24^.

Vortex pump vibrations are primarily periodic or quasi-periodic, with Cavitation often introducing oscillatory frequency characteristics. Frequency spectrum analyses, often performed via FFT methods, are critical for identifying vibration modes, critical speed, and the influence of flow conditions on vibration levels to prevent pump failures^25–27^.

Various cavitation detection techniques are reviewed in the literature, including vibration, pressure pulsation, and acoustic methods, each with unique strengths and limitations^20,28^.

Model-based approaches analysing voltage and current signals detect torque oscillations linked to hydraulic instabilities, employing advanced neural network architectures for early fault detection. A CNN-LSTM-attention network and a dynamic detection threshold are used in this method to detect abnormal operating conditions and early damage^29,30^.

This study addresses a significant gap knowledge regarding vibration thresholds capable of inducing resonance within water pumping stations. To remedy this shortcoming, the study focuses on characterizing vibrations caused by water vortices and pump component malfunctions. The problem of water vortices was addressed with a thorough approach. A comprehensive solution involving the construction of a specialized curtain wall inside the water channel. This novel strategy guarantees are enough to sustain peak performance that proposed and validated to effectively eliminate vortex formation, ensuring sustained peak performance through continuous root cause monitoring during station operation.

## Research methodology

Pumping stations are crucial to the supply of water for household, commercial, and agricultural uses. Typically, pumping stations are made to move water into a supply system from a water source, such a river.

**Fig. 1:**
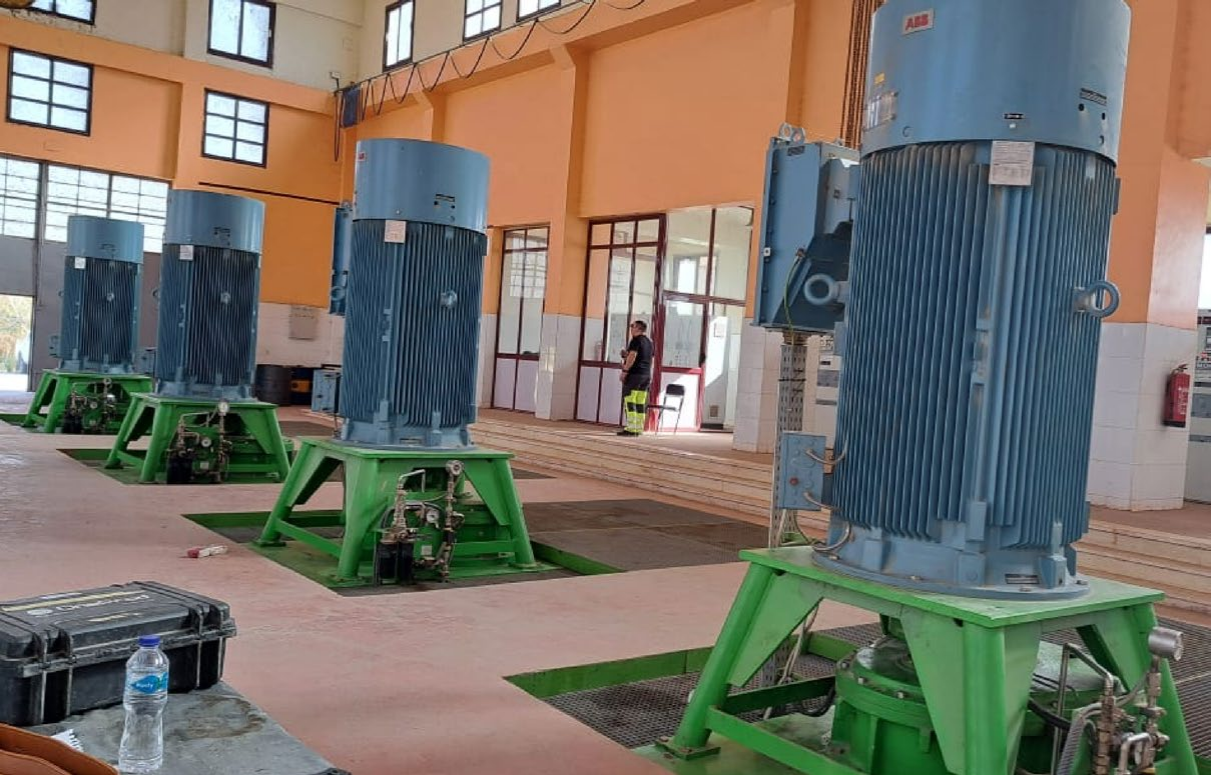
El-Marashda pumping station is situated in Ngea-Hammadi.

The El-Marashda pumping station is situated in Upper Egypt’s Ngea-Hammadi. The station is built on one of the Nile’s banks. The station, which irrigates 400 Km^2^, is made up of four mixed flow pump units. Four pumping units make up the station. Each pump has a design static head of 6.78 m and a discharge of 8 m^3^/s as shown in Fig. 1.

The river is where the water intake system is situated. During operation hours, the vibration levels across the pumping units of this pumping station were unstable and varied. For a continuous 6 h, the total vibration velocity level was recorded at four pumping units every 2 h. Velocity and acceleration spectra were analyzed at all measurement locations to investigate the extreme changes in vibration levels. Vibration levels fluctuated between the acceptable and unacceptable ranges. A visual investigation revealed that the top motor cover was vibrating more than usual. The main objective is to investigate the reasons behind variations of vibration levels throughout evaluation of pump units’ dynamic state.

**Fig. 2:**
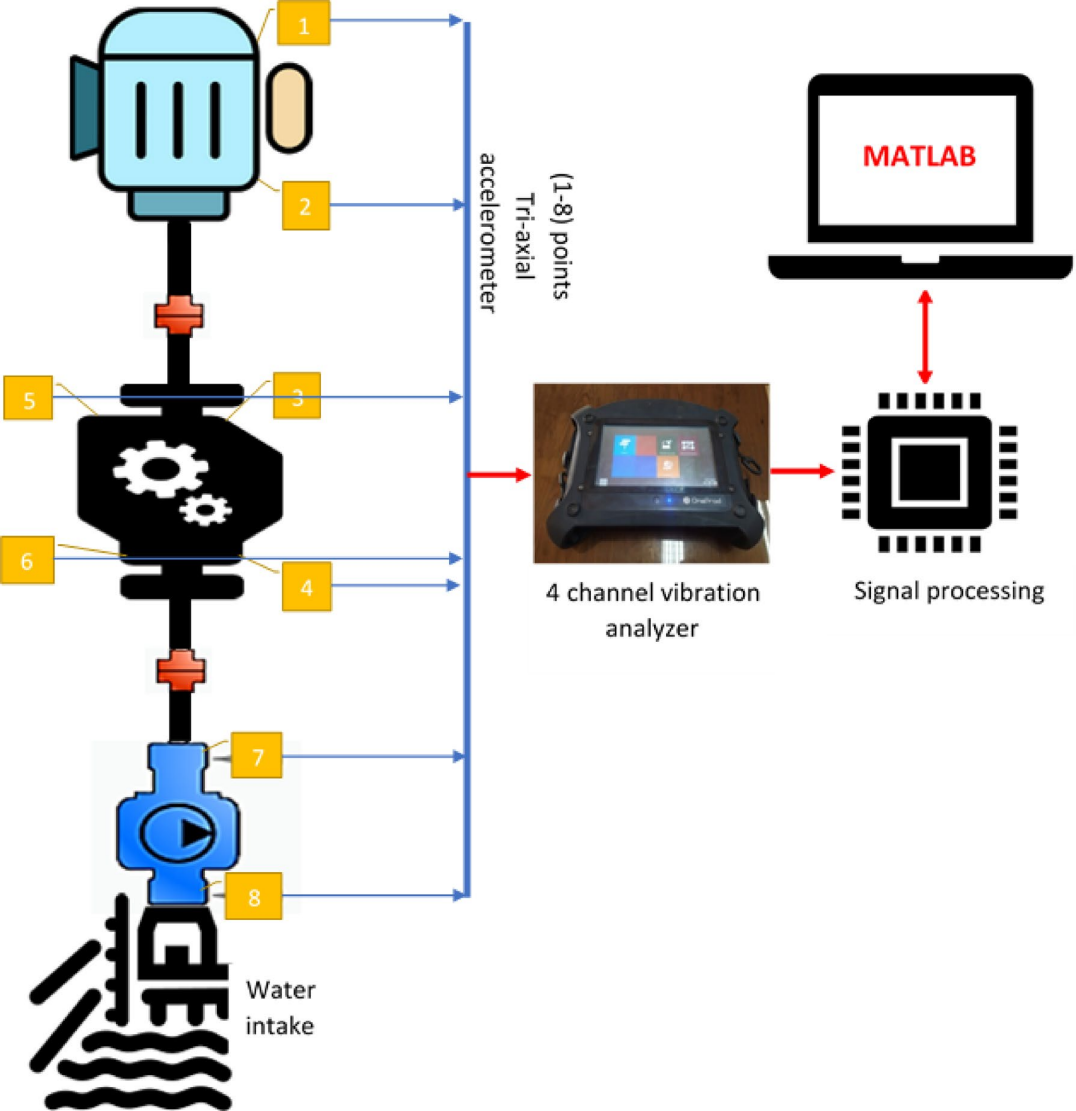
Layout of pumping unit and signal processing sequence.

Vibration accelerometers on the pumps are used to record vibration signals, and data is gathered at 15 preset measurement locations, as illustrated in Fig. 2. Two well-known methods were employed in the analysis to investigate the properties of the vibration signals: time–frequency representation and the Fast Fourier transform (FFT). To fully detect variations in the dynamic state, the vibration level of all four units was measured every 2 h for 6 h under the same operating condition.

Vibration measurements were done to specify the sources excitation operational frequencies at different locations. Measuring locations were chosen for the parts of the pumping unit that is highly sensitive to vibration, including the upper and lower bearings, and the pump thrust bearing at 9 locations in the axial, vertical, and radial directions. The signals from the accelerometers are directly fed into the analyzer which possesses an internal signal conditioning system comprising filters, integrators, amplifiers, etc. The signals are then transferred to the PC via USB connection to the software for signal analysis.

**Fig. 3:**
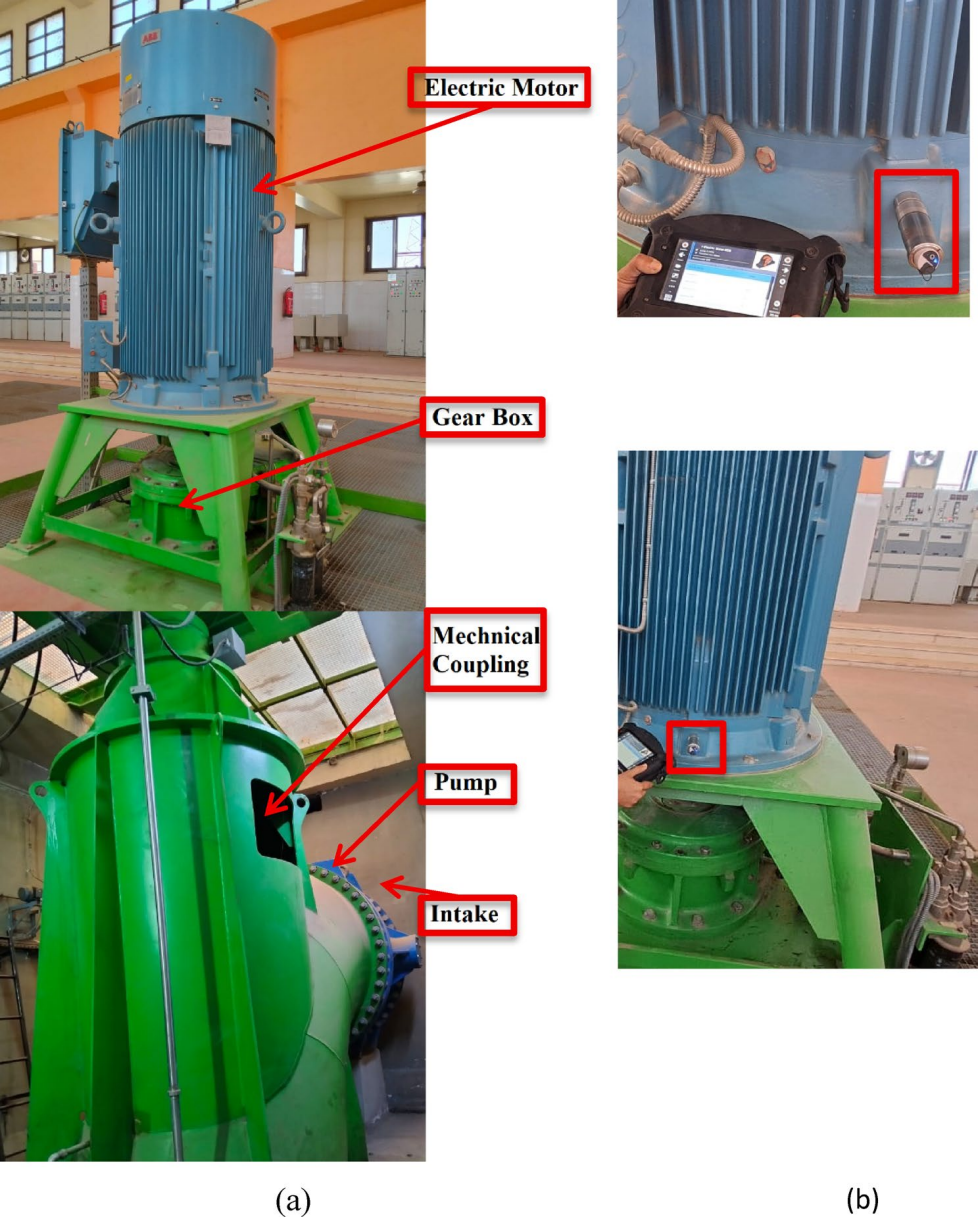
Measurement locations at pumping unit (**a**) setup pumping unit in two level vertical (**b**) installing of accelerometer on motor.

Frequency analysis of the resulting vibration spectrum was done through the relationship between velocity, acceleration, and frequency, which determines the sources of vibrations and their causes. To regulate the flaws that emerge in the medium and high frequency range, signals are measured in two frequency range groups: [0–1 kHz] and [2–20 kHz] in three different orientations (axial, horizontal, and vertical) as shown in Fig. 3a and b. In addition, temporal signals are measured and examined to identify any vibrations associated with shocks.

**Fig. 4:**
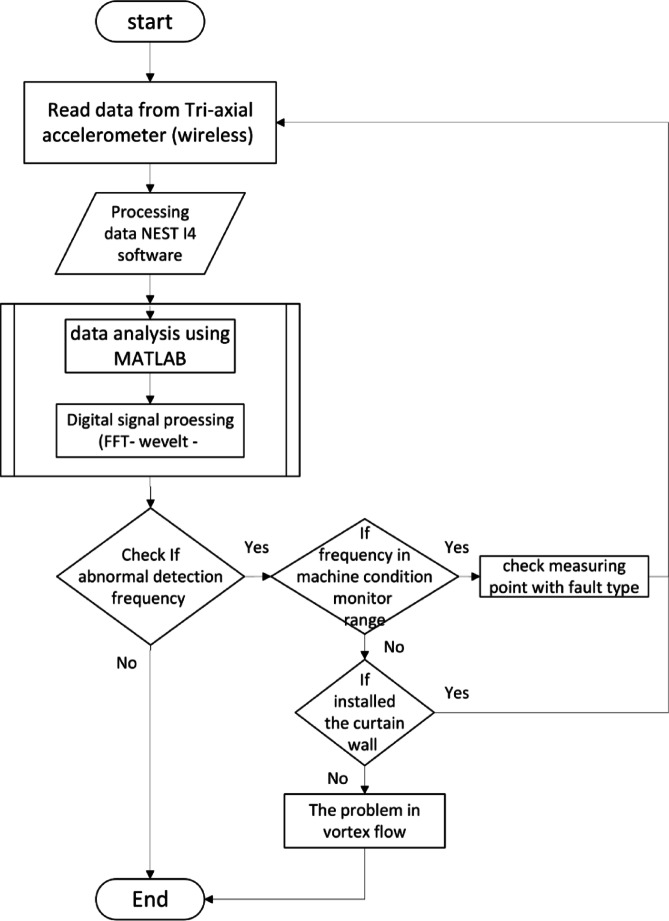
Operation algorithm methodology of vortex flow evaluation.

The research methodology employed a multi-step approach to evaluate vortex flow in an axial flow pump. Initially, wireless triaxial accelerometers were used to measure vibration frequencies. Subsequently, the collected signal data was preprocessed using NEST 14 software and classified into different frequency types through digital signal processing using MATLAB. This analysis enabled the identification of abnormal signals indicative of machine conditions or vortex flow as shown in Fig. 4.

The findings revealed that extreme oscillations in vibration levels were primarily attributed to hydraulic issues at the pump inlet, rather than underlying mechanical or electrical problems. Irregular shifts in the vibration patterns of the mechanical equipment served as a clear indicator of malfunctions. Abnormal vibrations in the pumping units further confirmed hydraulic problems.

## Measurements and vibration analysis

**Fig. 5:**
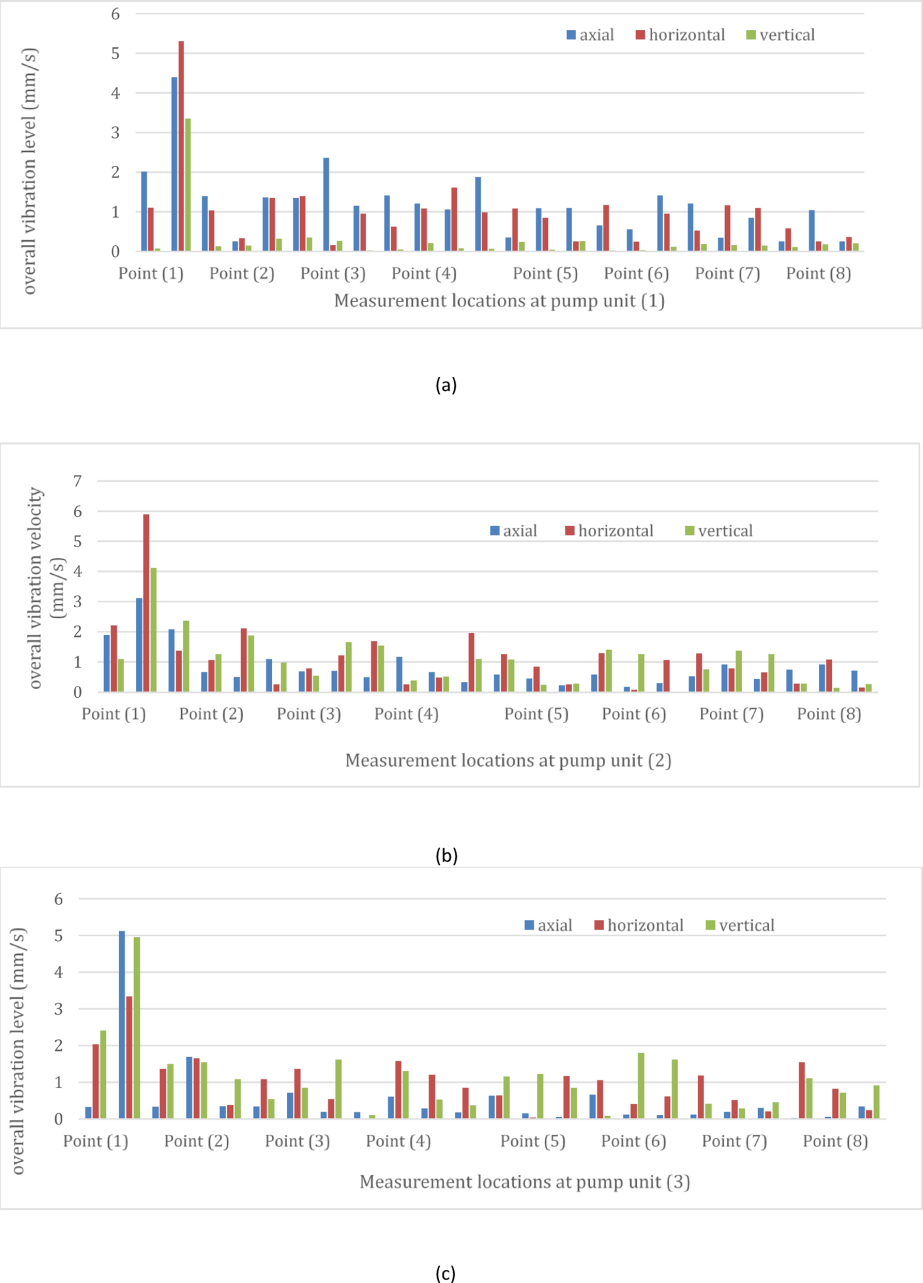

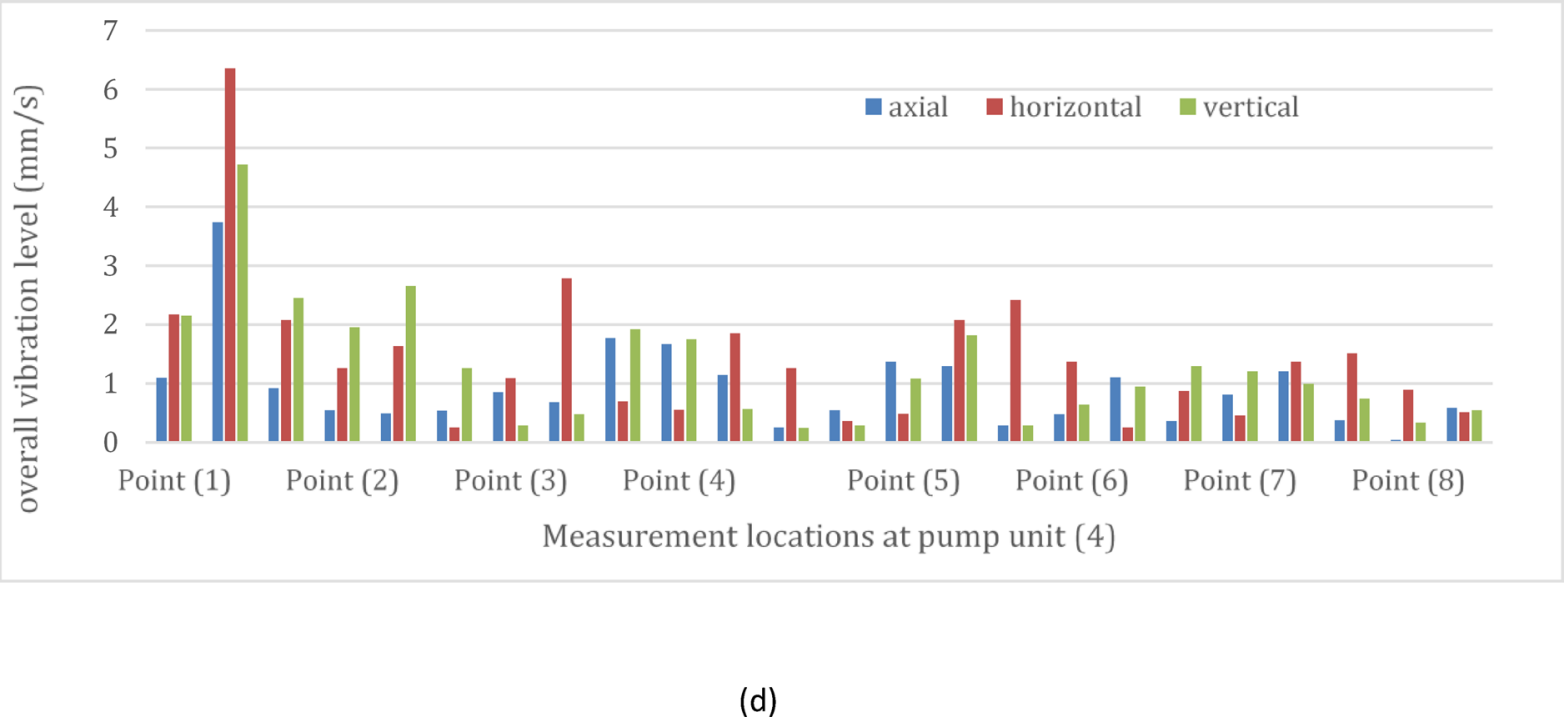
(**a**) Overall vibration velocity of pump unit (1) at a rate of every 2 h. (**b**) Overall vibration velocity of pump unit (2) at a rate of every 2 h. (**c**) Overall vibration velocity of pump unit (3) at a rate of every 2 h. (**d**) Overall vibration velocity of pump unit (4) at a rate of every 2 h.

The total vibration levels at the four pumping units, measured in three directions at a rate of every 2 h, were displayed in Fig. 5. It is evident that all units show fluctuating vibration levels over time, particularly at the motor’s non-drive end, which is identified as the weakest point on the pump. For the pump unit (1), overall vibration velocity changed within 6 h by increasing up to 58% and decreasing by 17%. Similarly, pump unit (2) experienced variations ranging from 89% increase to a 30% decrease. Pump unit (3) displayed overall vibration velocity fluctuations between 34% decrease and a 48% increase, while pump unit (4) showed variations from 70% increase to a 25% decrease.

**Fig. 6:**
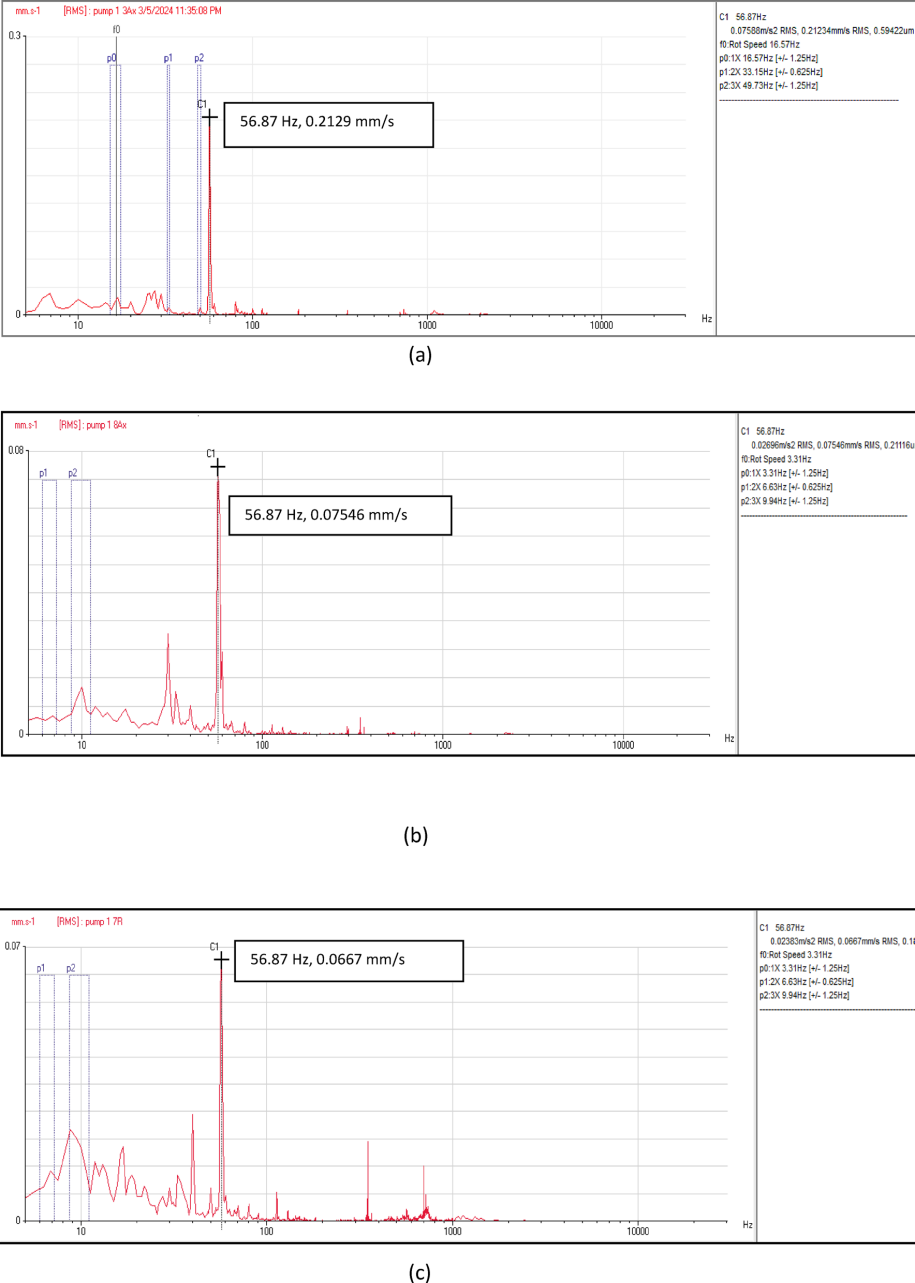

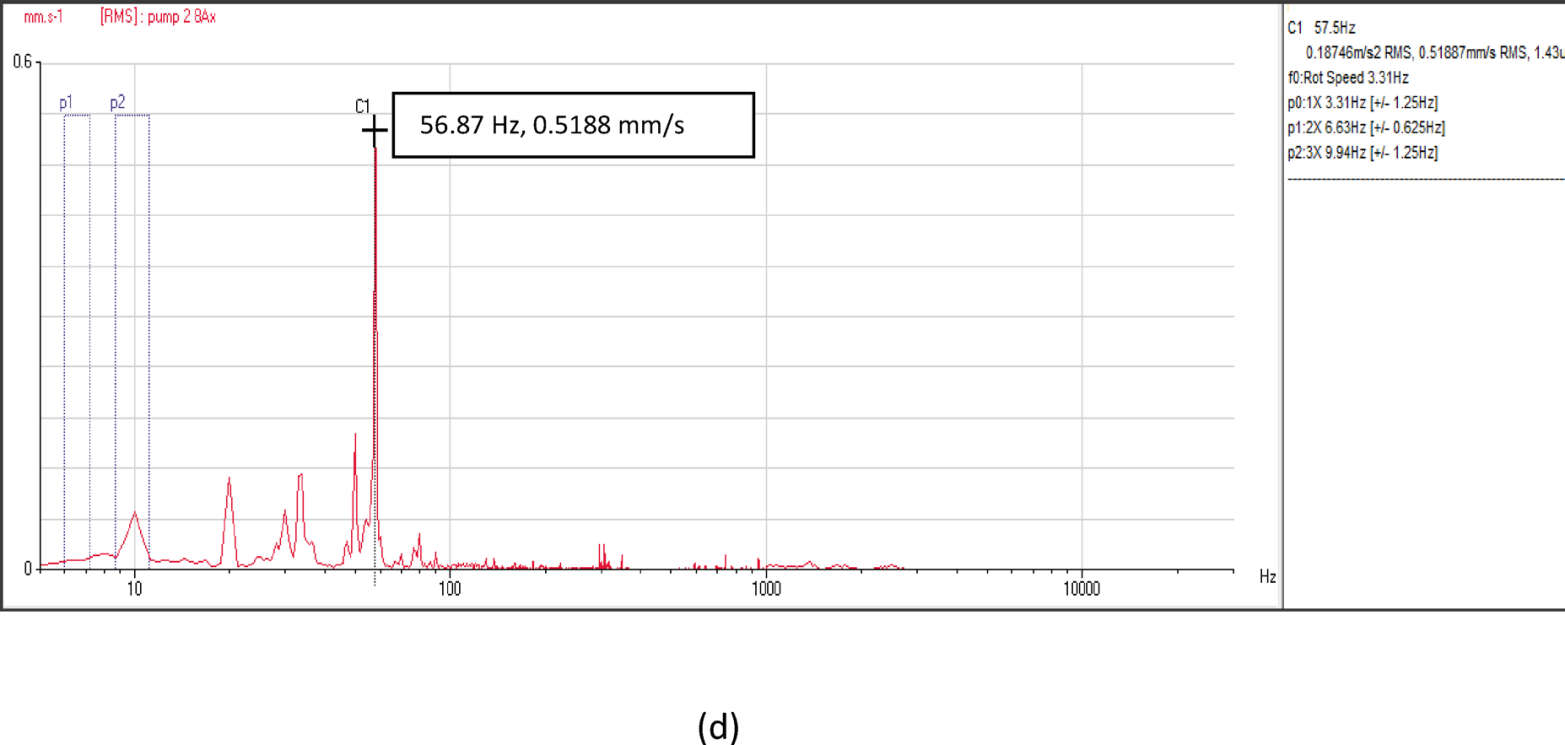
(**a**) Vibration velocity spectrum at pump unit (1). (**b**) Vibration velocity spectrum at pump unit (2). (**c**) Vibration velocity spectrum at pump unit (3). (**d**) Vibration velocity spectrum at pump unit (4).

Figure 6 depicts the vibration velocity spectra at the measurement points of the four pumping units. These signals include information related to flow instability faults, characterized by non-steady behavior in the frequency-velocity spectrum. Across all locations and directions—axial, horizontal, and vertical—a predominant peak was observed at the pump vane pass frequency (VPF) of 56.87 Hz, as illustrated in Fig. 6. Moreover, there is a notable minor peak in the low frequency range. The velocity spectra maintain similar patterns across all measurement points in the four units.

**Fig. 7:**
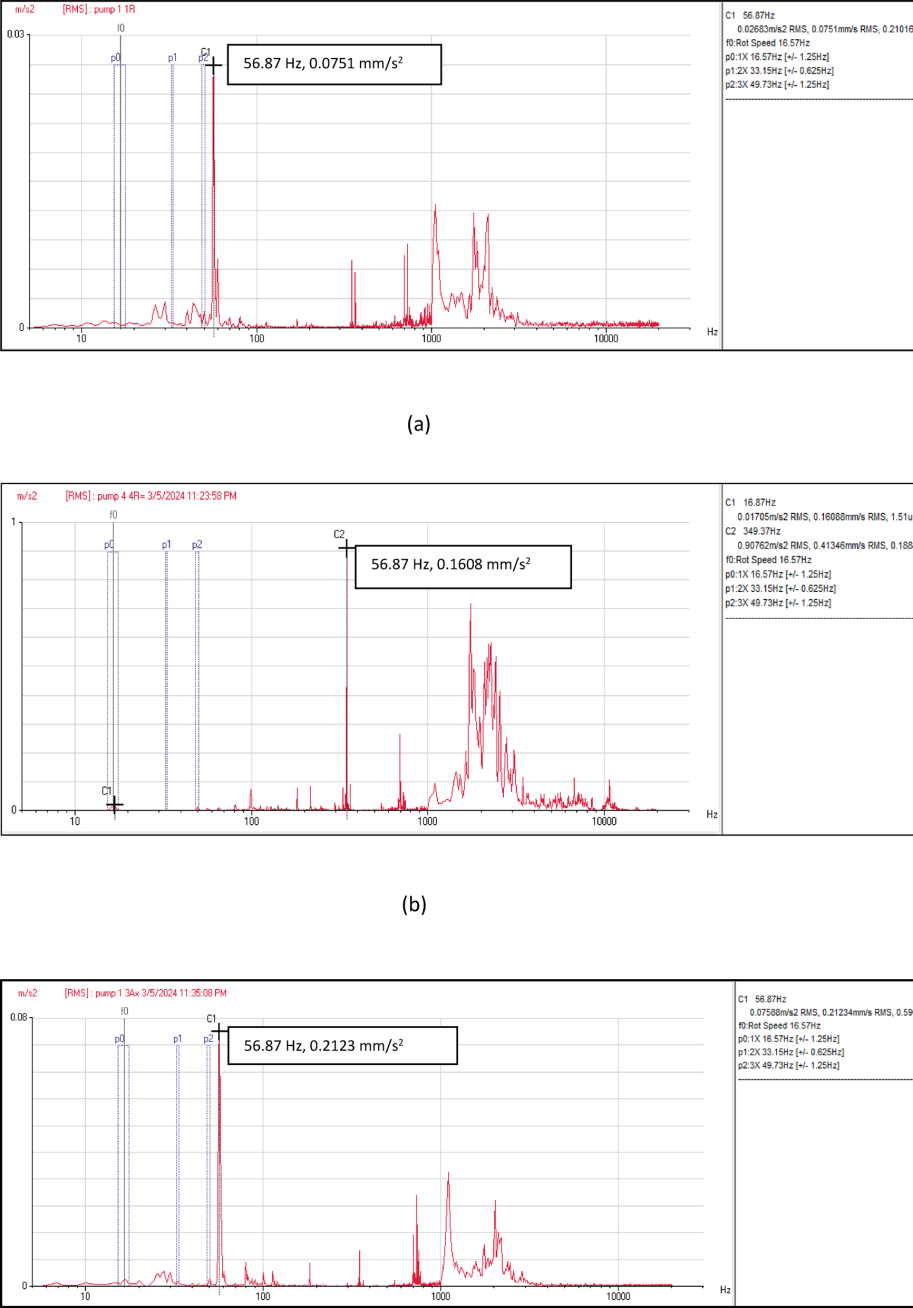

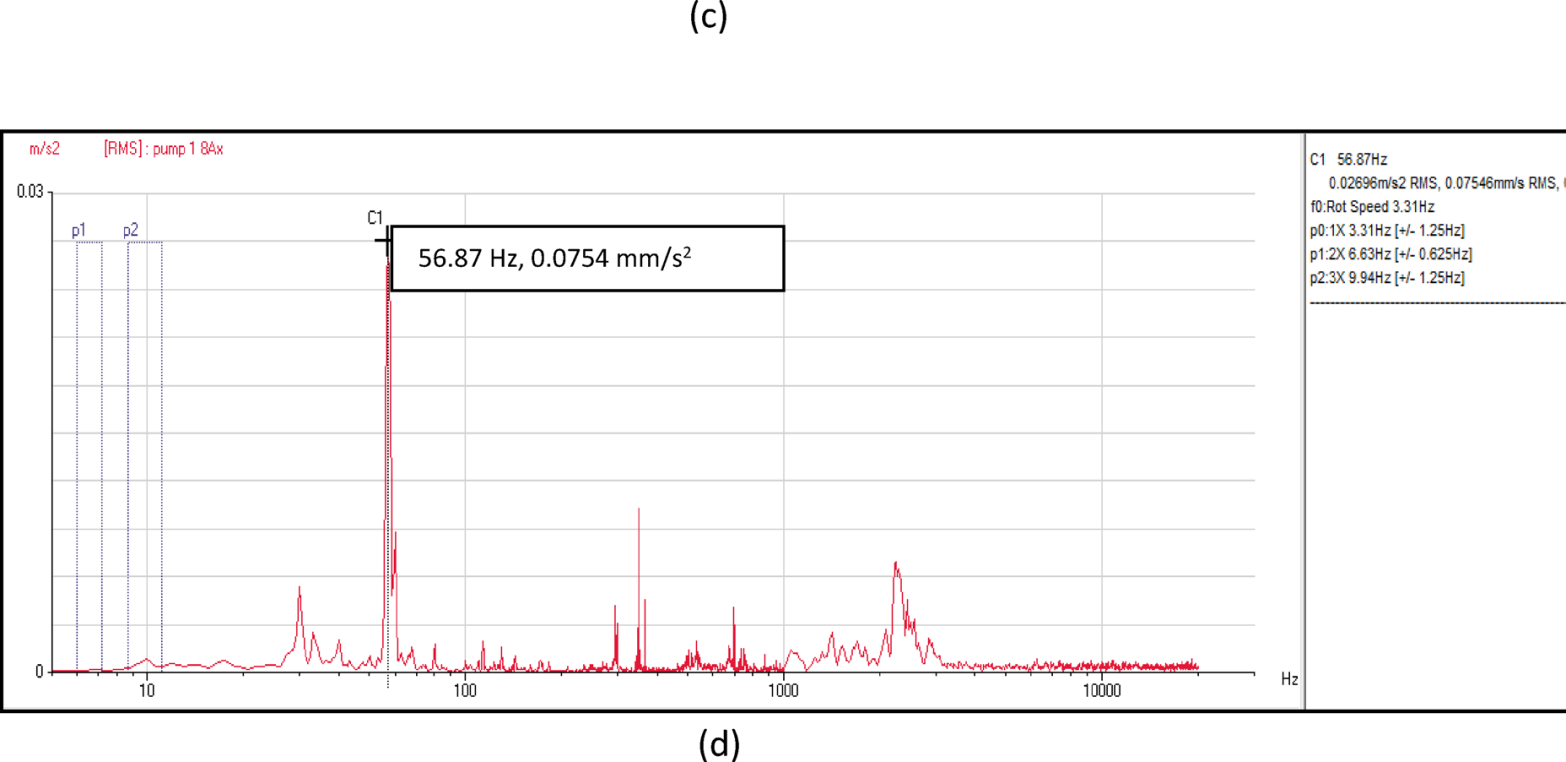
(**a**) Vibration acceleration spectrum at pump unit (1). (**b**) Vibration acceleration spectrum at pump unit (2). (**c**) Vibration acceleration spectrum at pump unit (3). (**d**) Vibration acceleration spectrum at pump unit (4).

Vibration acceleration signals at eight measurement locations at the four pumping units are collected for the same operating conditions as seen in Fig. 7. It is obviously seen that the vibration amplitude is affected by the flow rate in the frequency range of 1000–10000 Hz. The findings reveal a notable increase in frequency amplitude intensity within 1000–10000 Hz range that looks like a hump in the spectrum. Also, there is a distinct peak at vane pass frequency (VPF) at all acceleration spectrum that measured at all locations of the four pumping units.

**Fig. 8:**
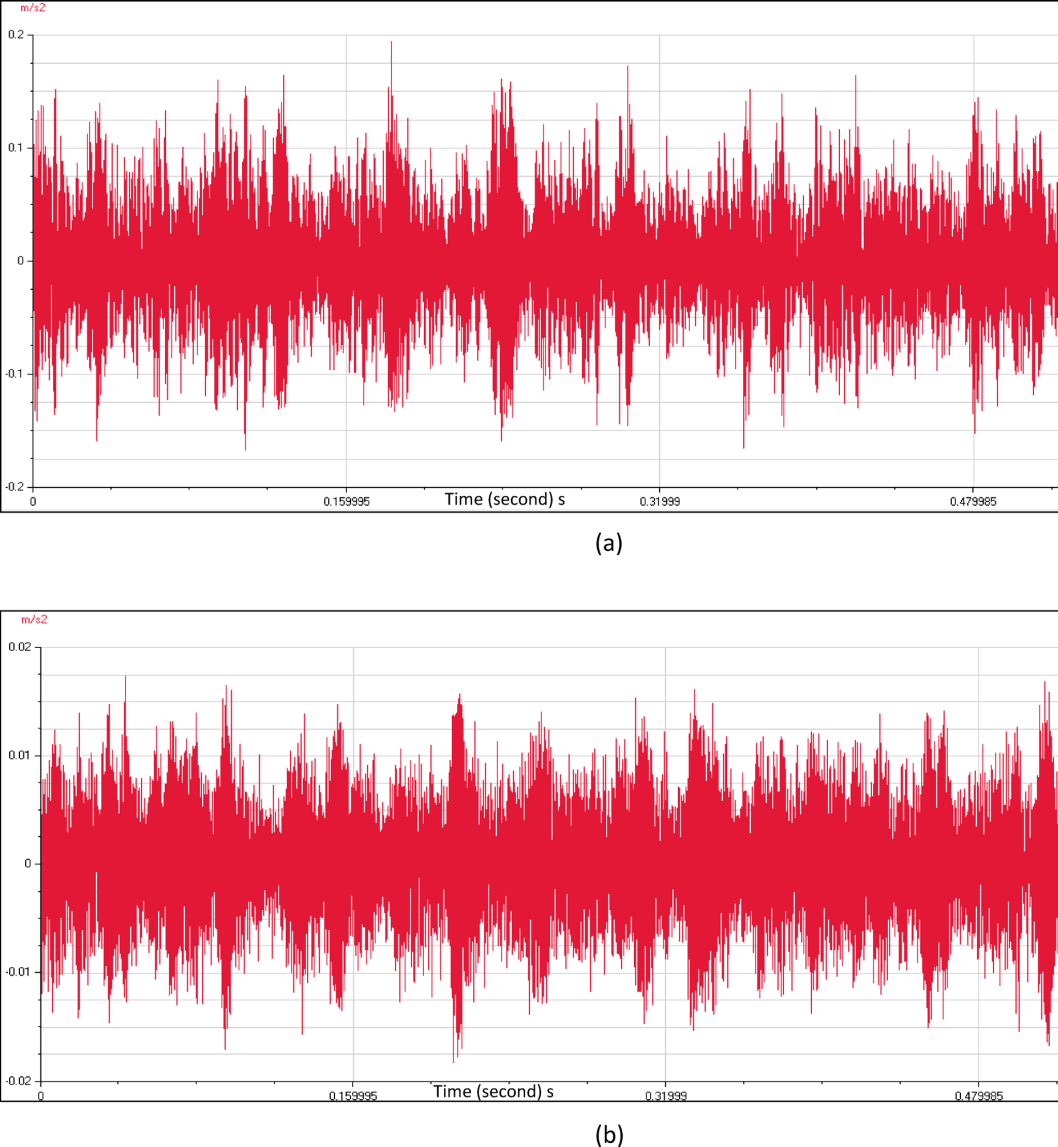

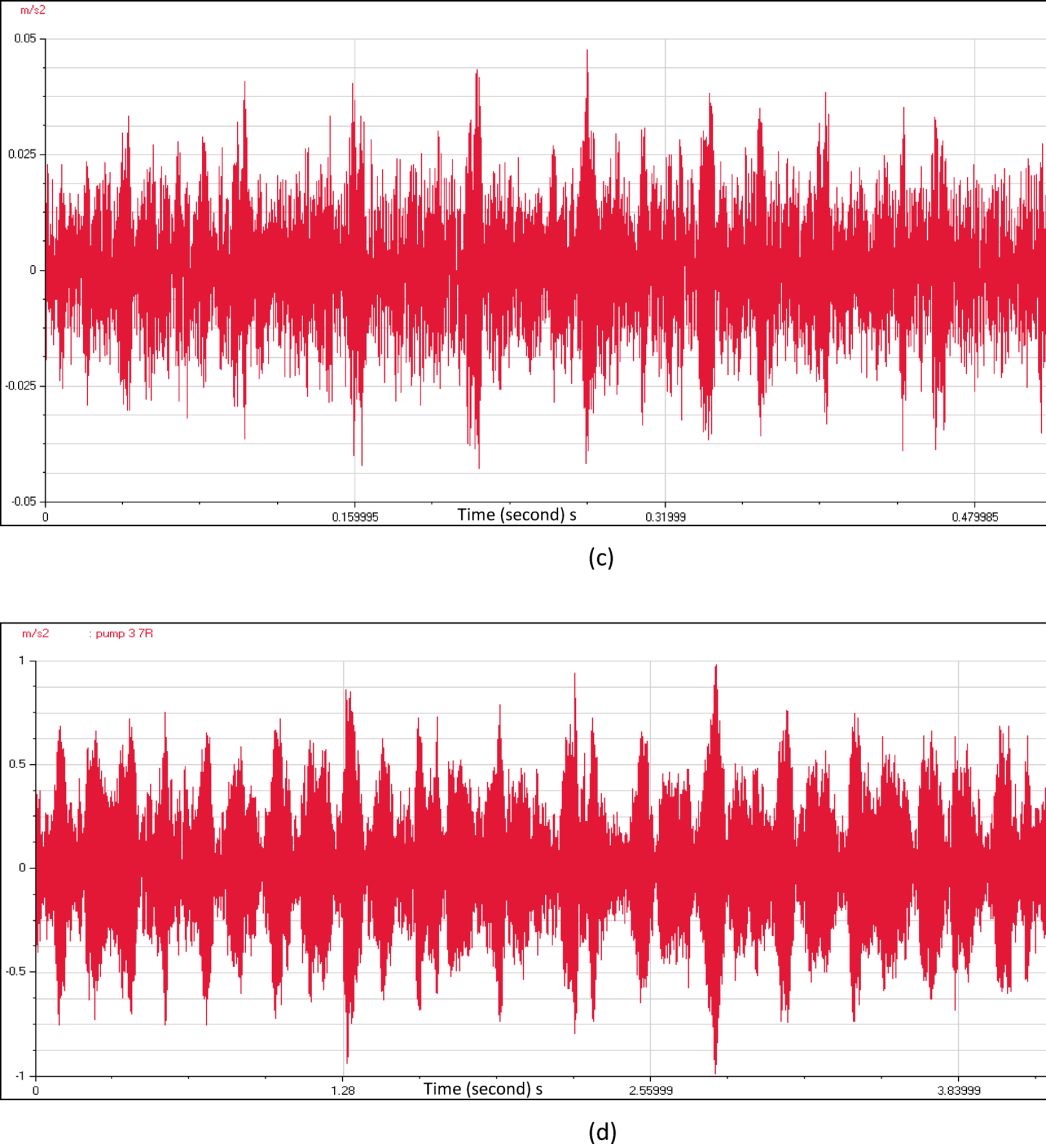
(**a**) Time wave form measured at pump unit (1). (**b**) Time wave form measured at pump unit (2). (**c**) Time wave form measured at pump unit (3). (**d**) Time wave form measured at pump unit (4).

Figure 8 shows the time wave form characteristics of the four pumping units as a relation between time (seconds) and acceleration (m/s^2^). The close insight indicated many pulsations which are not evenly spaced. The source of these pulsations is not periodic, and it is normally creating random high frequency vibrations. These time waveforms contain key details related to pump flow problems. As a result, it is necessary to apply a numerical simulation of the pump station intake to investigate the main causes of the flow instabilities.

## Numerical modelling analysis

It is important to illustrate that the pumps are working in smooth intakes, with the swirl-free flow at their entrance. The pump’s intakes and the channels that surround the pump bells are only experimentally designed. Experimental design always relies on that study applied to lab prototypes and experience gained from installations. CFD analysis achieves the goal of deep insight and improving the realization of the flow and its characteristics in the sump. The uniform flow could be tested depending on its distribution in the pump intake. This is particularly important to discover the causes of vortex formation more accurately by using CFD, Kushwaha^31^.

The design of the pump intake is especially important to ensure proper flow and prevent cavitation. Proper pump intake design must be considered to minimize flow obstructions and sudden changes in flow direction and provide adequate submergence. The intake should be located away from the pump discharge to avoid recirculation.

Poor pump intake design can lead to harmful issues like cavitation, air entrainment, vortex, and uneven flow distribution that can negatively impact pump performance and reliability.

In the present study, the CFD software FLUENT, which utilizes the finite element scheme for non-structured meshes, is used for solving the steady state Reynolds-Averaged Navier–Stokes (RANS) equations, Gülich and Friedrich^32^. The equations in the two-phase flow in the Cartesian coordination system (xi), with velocity components (ui) where i = 1, 2, 3, are expressed as:

1 $${V}_{F}\frac{\partial \rho }{\partial t}+ \frac{\partial }{\partial {x}_{j}} \left(\rho {u}_{i}{A}_{i}\right)=0$$

2 $$\frac{\partial {u}_{j}}{\partial t}+ \frac{1}{{V}_{F}}\left\{{u}_{j}{A}_{i}\frac{\partial {u}_{i}}{\partial {x}_{j}}\right\}= -\frac{1}{\rho }\frac{\partial p}{\partial {x}_{i}}+{G}_{i}+{f}_{i}$$

where V_F_ is the fractional volume open to flow, ρ is the water density, with A_i_ representing the fractional area open to flow in (i) direction. In Eq. (2), p denotes pressure, while (G_i_) and (f_i_) represent the body and viscose acceleration. In this study, a three-dimension simulation is done for the intake of a mixed flow type water pump with discharge 8 m^3^/s, and static head 6.78 m however, the rotational speed of the pump is 199 RPM. The type of casing for the impeller is a diffuser type and the type of the impeller is an open and fixed blade type. Simulation the numerical modeling was carried out only for the discharge conduit of the pump, without including the rotating impeller domain. Therefore, a rotor–stator interface was not required in the simulation setup. Regarding turbulence modeling, the Shear Stress Transport (SST) k-ω model was employed since it has been widely validated in literature for internal flows with strong curvature and possible flow separation. This model provides a good compromise between accuracy and robustness for predicting pressure losses and velocity distribution inside the discharge pipe. A fine grade generation mesh is built using the mesh modeler program with an element size of 5 mm, which is a good sizing according to Pandey et al.^33^.

A high-quality computational mesh was generated using a hybrid approach combining structured hexahedral elements in the main flow domain with unstructured tetrahedral elements in the complex regions. Special attention was given to resolving the near-wall flow behavior by introducing inflation layers along the solid boundaries. In total, inflation layers were applied with a growth rate of 1.2, ensuring a smooth transition from the wall to the core flow.

The simulations were conducted using the finite volume method (FVM) with the turbulence model selected for this study was the Shear Stress Transport (SST) k-ω model, which requires fine near-wall resolution. Therefore, the mesh was refined to achieve a dimensionless wall distance (y^+^) of approximately 1 across the majority of the wall surfaces. This resolution enables direct integration through the viscous sub-layer without the use of wall functions, ensuring accurate prediction of boundary layer separation and wall shear stresses. The boundary layer mesh has been defined using a structured prism layer to resolve the near-wall region. It consists of 10 inflation layers (growth rate of 1.2) with a first-layer thickness set for y^+^ ≈ 1. This setup provides the necessary near-wall resolution for the SST k-ω turbulence model to accurately predict wall shear stress and pressure gradients. To verify mesh adequacy, three computational grids were generated to verify the grid independence of the numerical results, containing 1.52 million (coarse), 2.75 million (medium), and 3.43 million (fine) elements, respectively. The monitored parameters were the discharge Q, head H, and hydraulic efficiency η. As shown in Table 1, the differences between the medium and fine meshes were less than 0.2% for both Q and H, and 0.3% for η. Therefore, the medium mesh was selected for the remaining simulations as it provided mesh-independent results with reasonable computational cost. The global mass imbalance remained below 0.5%, ensuring solution consistency.

**Table 1:** Grid independence study.

Mesh level	Number of elements	(Q_{num}) (m^3^/s)	ΔQ versus previous (%)	(H_{num}) (m)	ΔH versus previous (%)	(η_{num}) (%)	Δη versus previous (%)	Mass imbalance (%)
Coarse	1,523,487	7.78	–	6.60	–	80.0	–	0.42
Medium	2,754,164	7.85	+ 0.90%	6.65	+ 0.76%	80.7	+ 0.88%	0.31
Fine	3,425,617	7.86	+ 0.13%	6.66	+ 0.15%	80.9	+ 0.25%	0.28

Boundary and initial conditions, at the inlet, a mass-flow (or velocity) boundary condition was prescribed to match each experimental operating point Q. Turbulence quantities were initialized via standard correlations based on the hydraulic diameter D as a following equation.

3 $$mass\;flow\;rate= \rho A U$$

4 $$0.07 D={Re}^{-1/8}$$

5 $$0.16 l=I$$

where A is the inlet cross-sectional area and U is the is the area-averaged inlet velocity. However, I is turbulence intensity $l$ the turbulence length scale and Re is the Rynolds number^34,35^.

Pressure–velocity coupling used SIMPLE (or Coupled, if stated), with second-order discretization for pressure and momentum (and turbulence equations). Gradients were reconstructed with a Green–Gauss node-based method. Convergence criteria required scaled residuals < **1 × 10**^**−5**^ for all equations and a global mass imbalance < **0.5%**. Area-averaged outlet flow and integral pressure drop were monitored to ensure steady asymptotic behavior.

The numerical results have been validated against available experimental data of the actual input and outlet flow at the intake sump and discharge pipe. The validation was carried out by comparing the simulation results with the measured data at corresponding operating points.

The relative error (RE) between the numerical prediction and the experimental measurement was calculated using the following expression:

6 $$RE\%=\frac{{X}_{num}-X}{X} \times 100$$

where X_num_ is the numerical result and X is the actual result from experimental measurements. Summarizes the comparison between CFD and experimental data. The maximum deviation in head and flow rate was less than **5%**, while the deviations in power and efficiency remained below **8%**, indicating good overall agreement, which is within the acceptable range reported in the CFD literature for turbomachinery simulations. This validation confirms the reliability of the numerical setup and supports the conclusions of the present study.

**Fig. 9:**
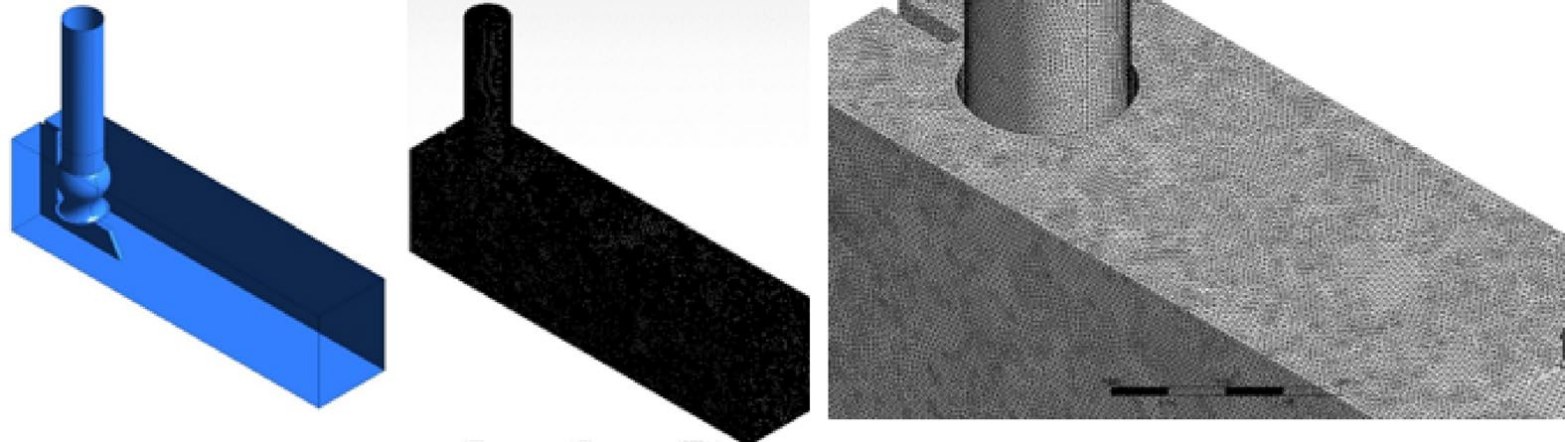
full dimension 3-d model and mesh structure of the pump intake, created using ANSYS^37^.

The detailed visualization in Fig. 9 illustrates the 3D vorticity structures within the basin and around the pipe, utilizing Q = 5 iso-surfaces. The presence of a free surface vortex located between the pump and basin wall, also a corner vortex, is distinctly observed. Figure 9 also depicts the full-scale 3D model and mesh configuration of the pump intake.

**Fig. 10:**
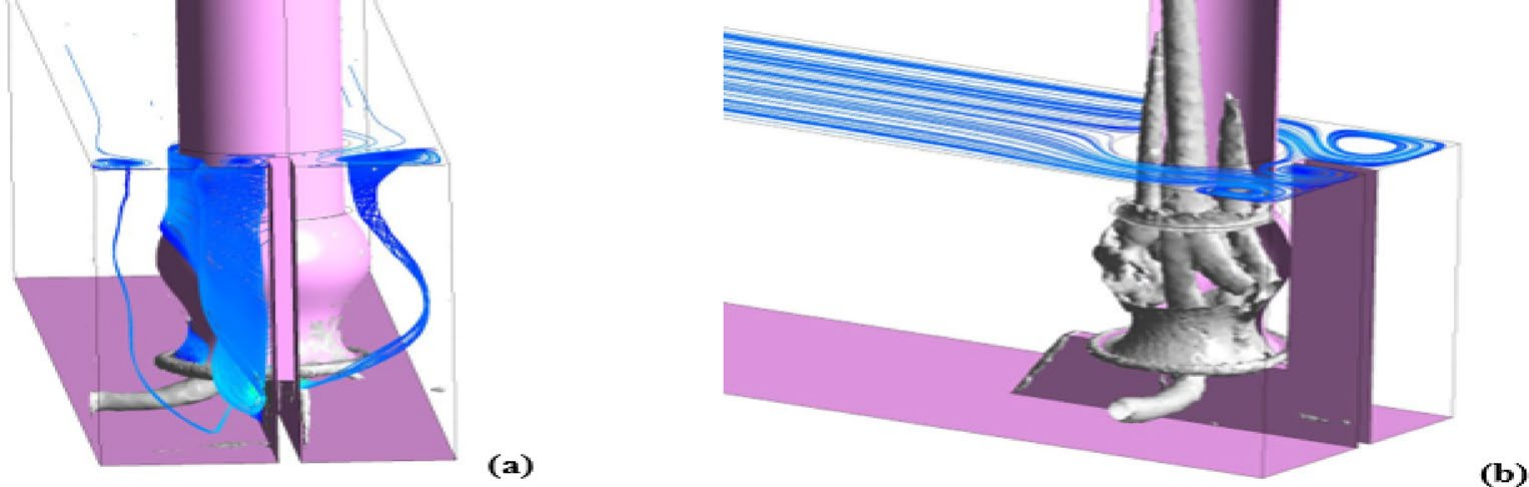
3D vorticity and streamline (**a**) 3D vorticity inside the suction line (**b**) of the present design, created using ANSYS^37^.

Beyond the free surface vortex, several subsurface vortices are identified. These vertices originate from the side walls and extend toward the pipe’s center, as shown in Fig. 10a. Additionally, several smaller vortices form inside the pipe itself, as illustrated in Fig. 10b. Also, the rotation axes of these vortices are aligned parallel to the adjoining wall.

**Fig. 11:**
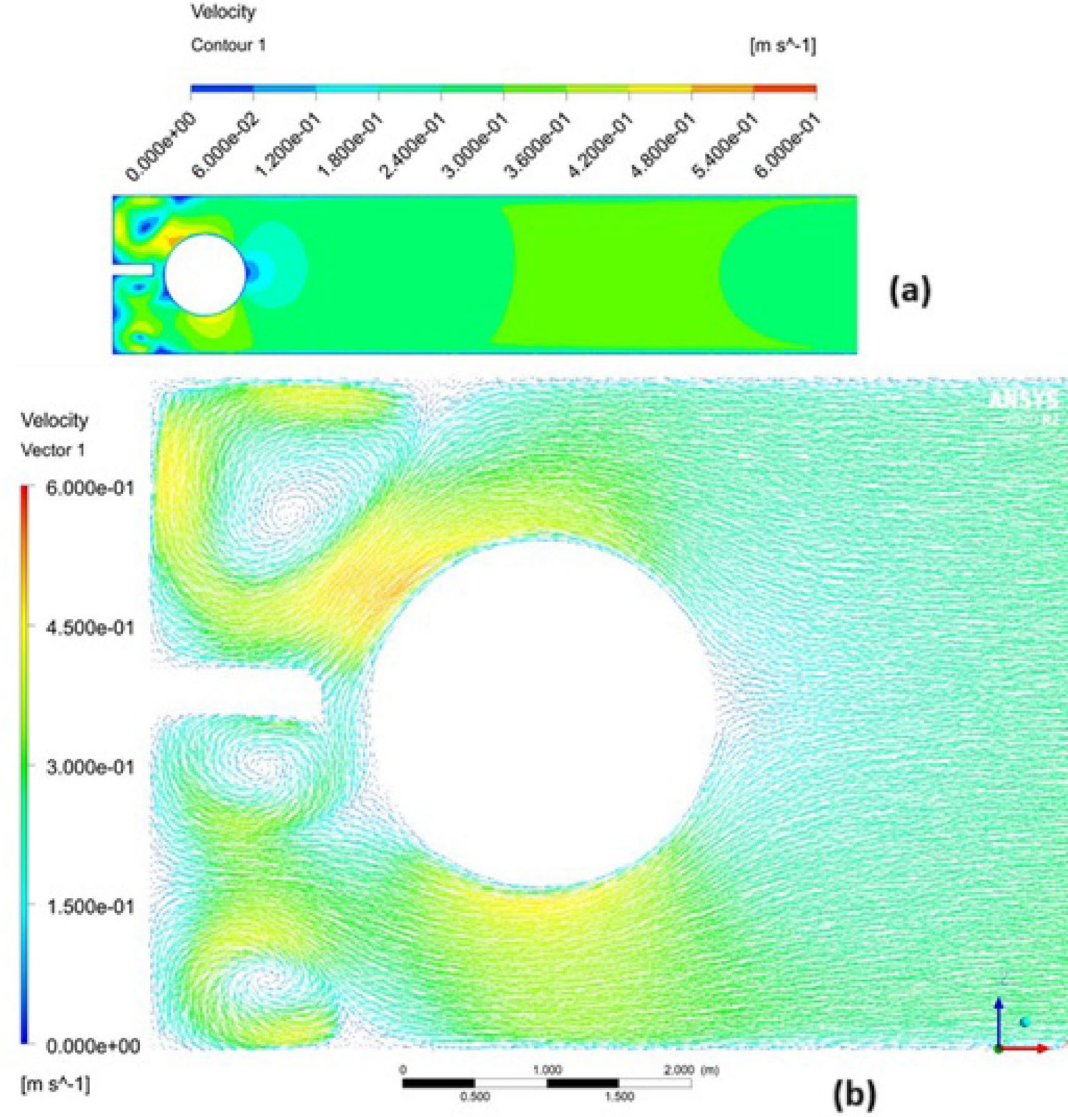
Velocity (**a**) Vector (**b**) vector contour at the surface layer, created using ANSYS^37^.

The free surface vortices could be characterized through velocity and vector contour at the surface layer, as shown in Fig. 11. Velocity magnitude within the vortex-affected surface layer fluctuates significantly, ranging from 0 to 0.6 m/s over localized areas, as indicated in Fig. 11a. Correspondingly, the flow direction exhibits variability along the free surface layer, which is clearly depicted by the vector contours in Fig. 11b, indicating flow direction fluctuations particularly pronounced within the vortex zones.

**Fig. 12:**
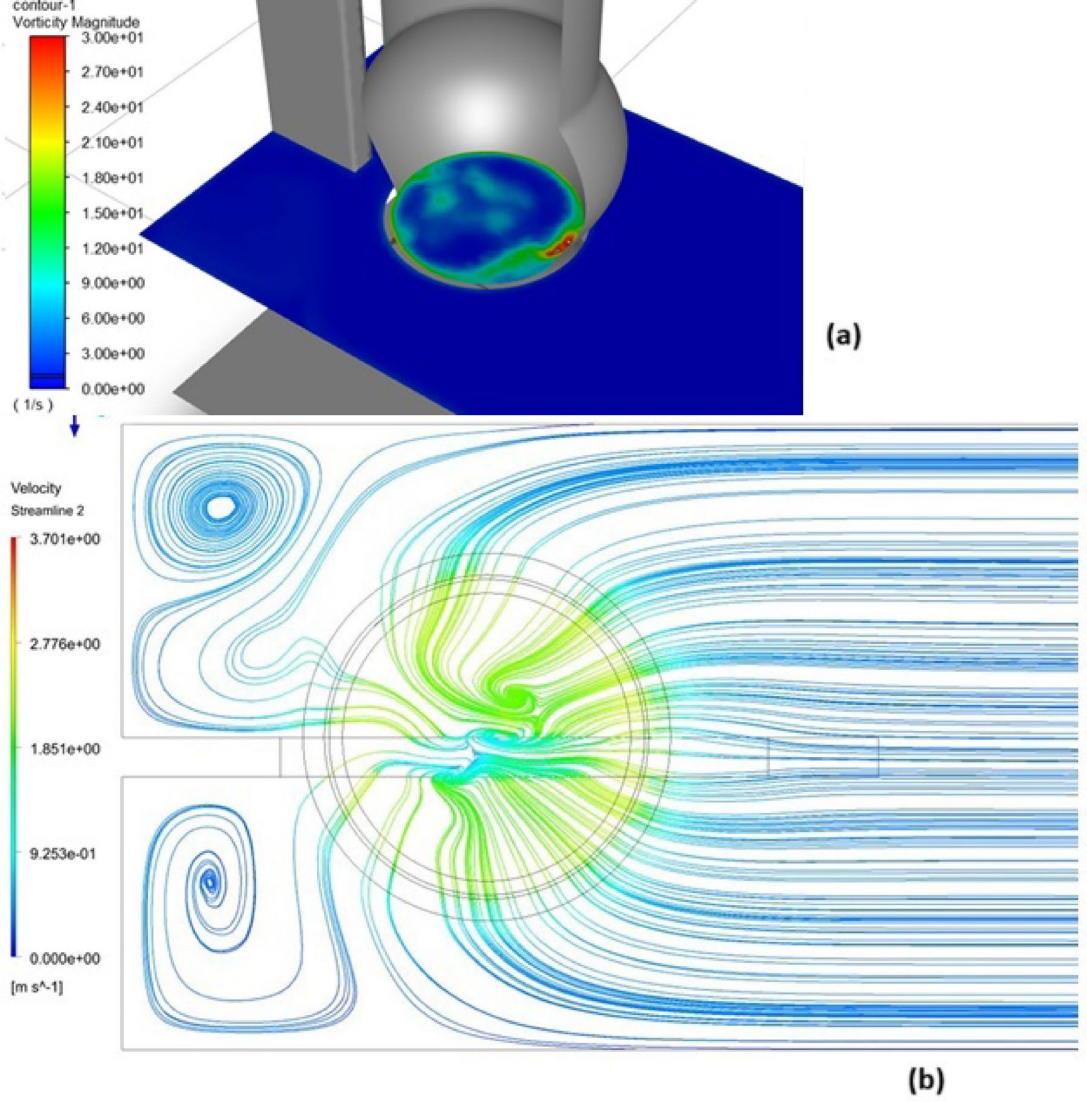
The vortices (**a**) and streamline (**b**) at the suction pipe entrance, created using ANSYS^37^.

Figure 12 presents the streamline behavior within the vortex region, confirming the existence of several critical vortices near the pipe center. The maximum vorticity magnitude observed approaches 15 degrees, underscoring the critical nature of flow conditions at the pipe entrance. The swirl angle, or vorticity magnitude, serves as a key parameter in assessing pump efficiency. Based on the hydraulic model studies, reported by Anvar^36^ recommended that the maximum acceptable magnitude of the swirl angle is 5°. Figure 12a and b showed the vorticity magnitude contour and streamline performance at the pipe entrance, respectively, further emphasizing the need to control vortex intensity for optimal pump performance.

**Fig. 13:**
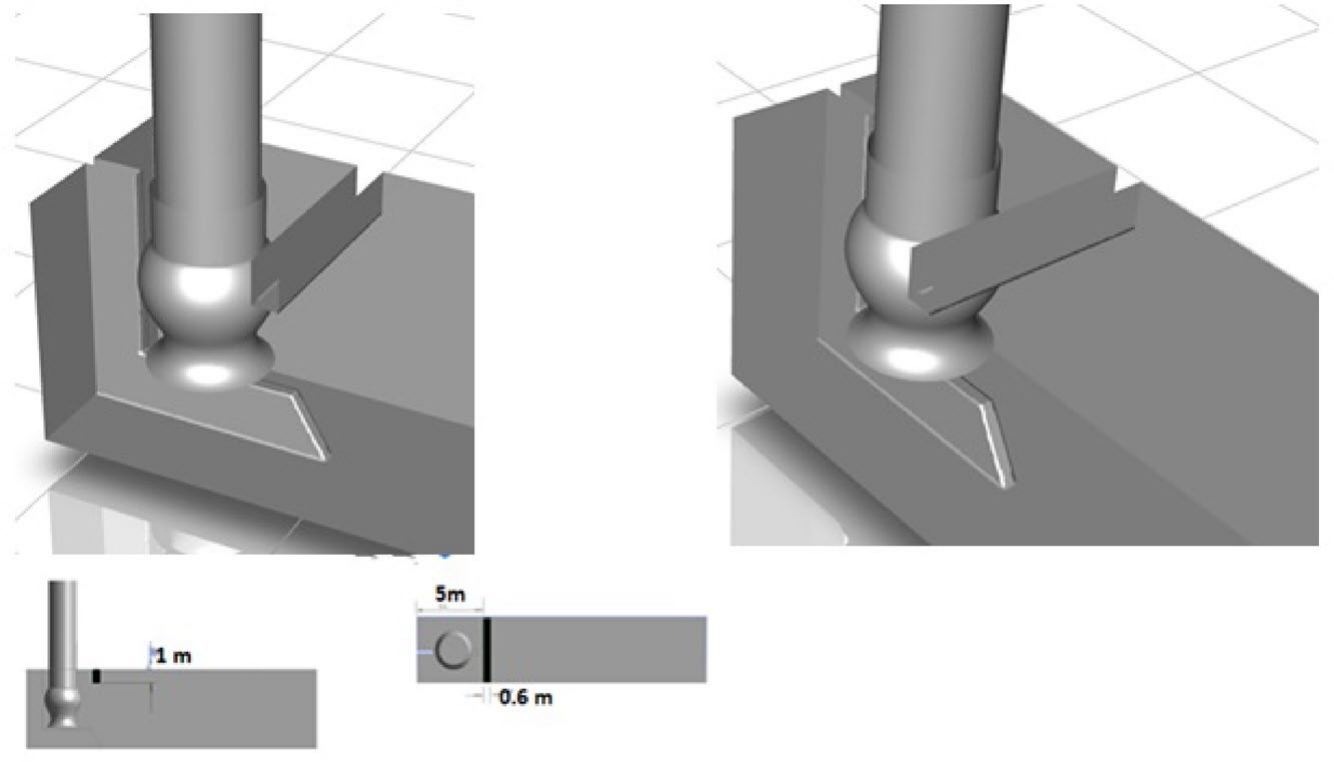
Recommended design of pump intake, created using ANSYS^37^.

Due to the available references for designing the pump intakes, distinct types of anti-vortex devices are used to reduce the intensity of free surface, subsurface, and floor-attached vortices. In the present study, a few simulations have been conducted to examine the use of side and corner fillets, as well as the curtain wall, as common measures for mitigating or eliminating free surface and subsurface vortices. The recommended design of the pump intake after adding the curtain wall is shown in Fig. 13.

**Fig. 14:**
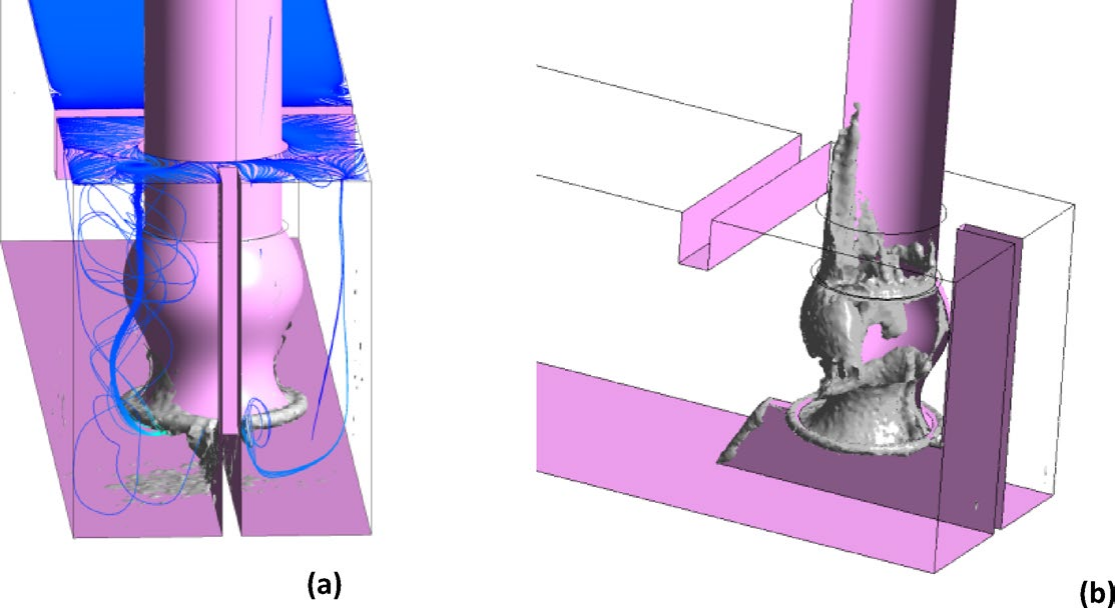
3D vorticity and streamline (**a**) 3D vorticity inside the suction line (**b**) of recommended design, created using ANSYS^37^.

Figure 14, which is displayed by comparing with Fig. 10, depicts the 3D vorticity structures in the basin and surrounding the pipe using Q = 5 iso-surfaces. The free surface vortex between the pump and the wall and the corner vortex completely disappeared. The free surface, behind wall, and center line of pipe vortices was decreased due to the recommended design.

## Results and discussions

**Fig. 15:**
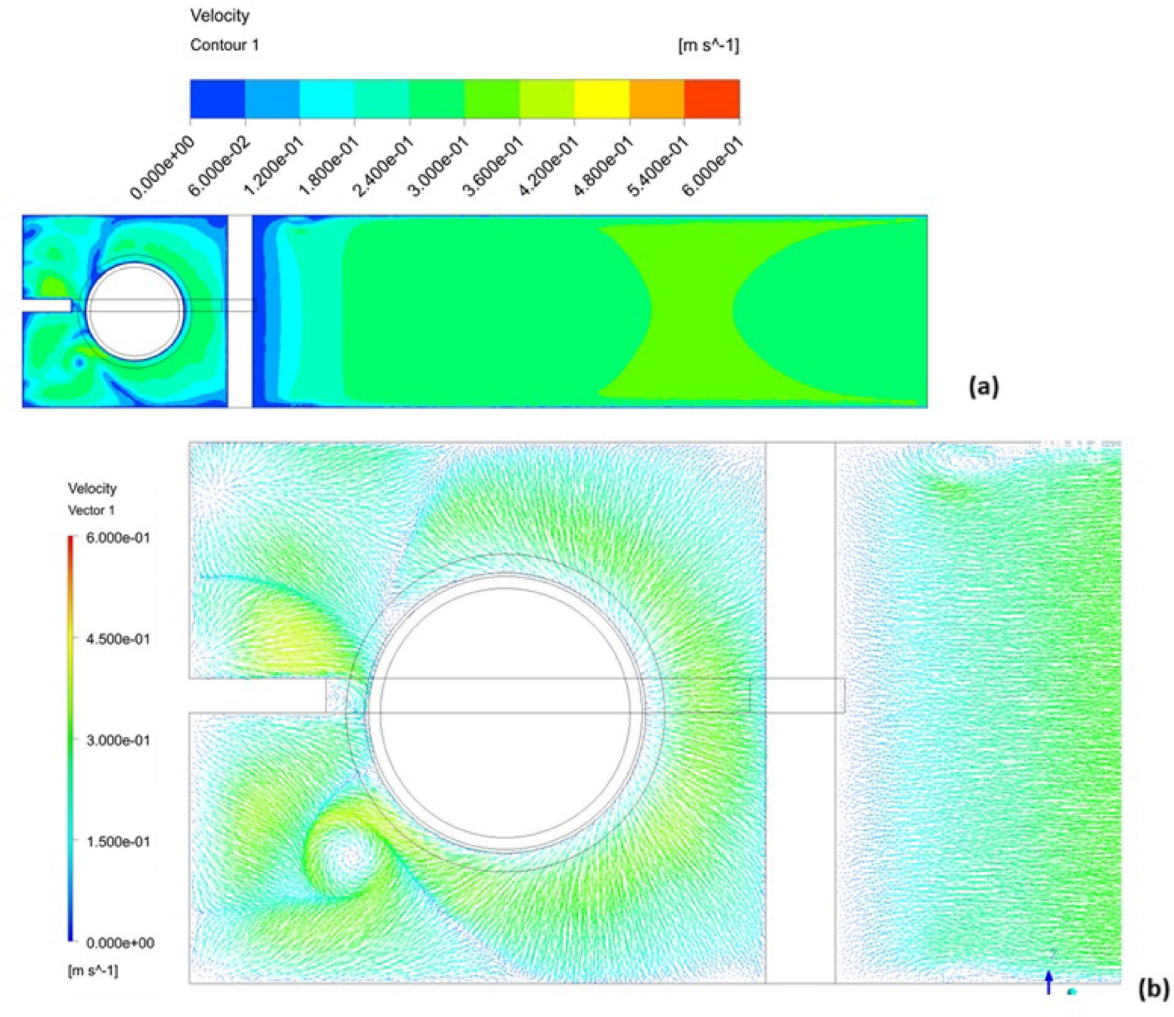
Velocity (**a**) vector (**b**) contour at the surface layer of recommended design, created using ANSYS^37^.

The performance of the flow at the surface layer was improved as seen in Fig. 15a and b. The velocity contour presented a good distribution of the velocity magnitude compared with the previous case. Whenever direction of the flow showed that the free surface vortices were eliminated.

**Fig. 16:**
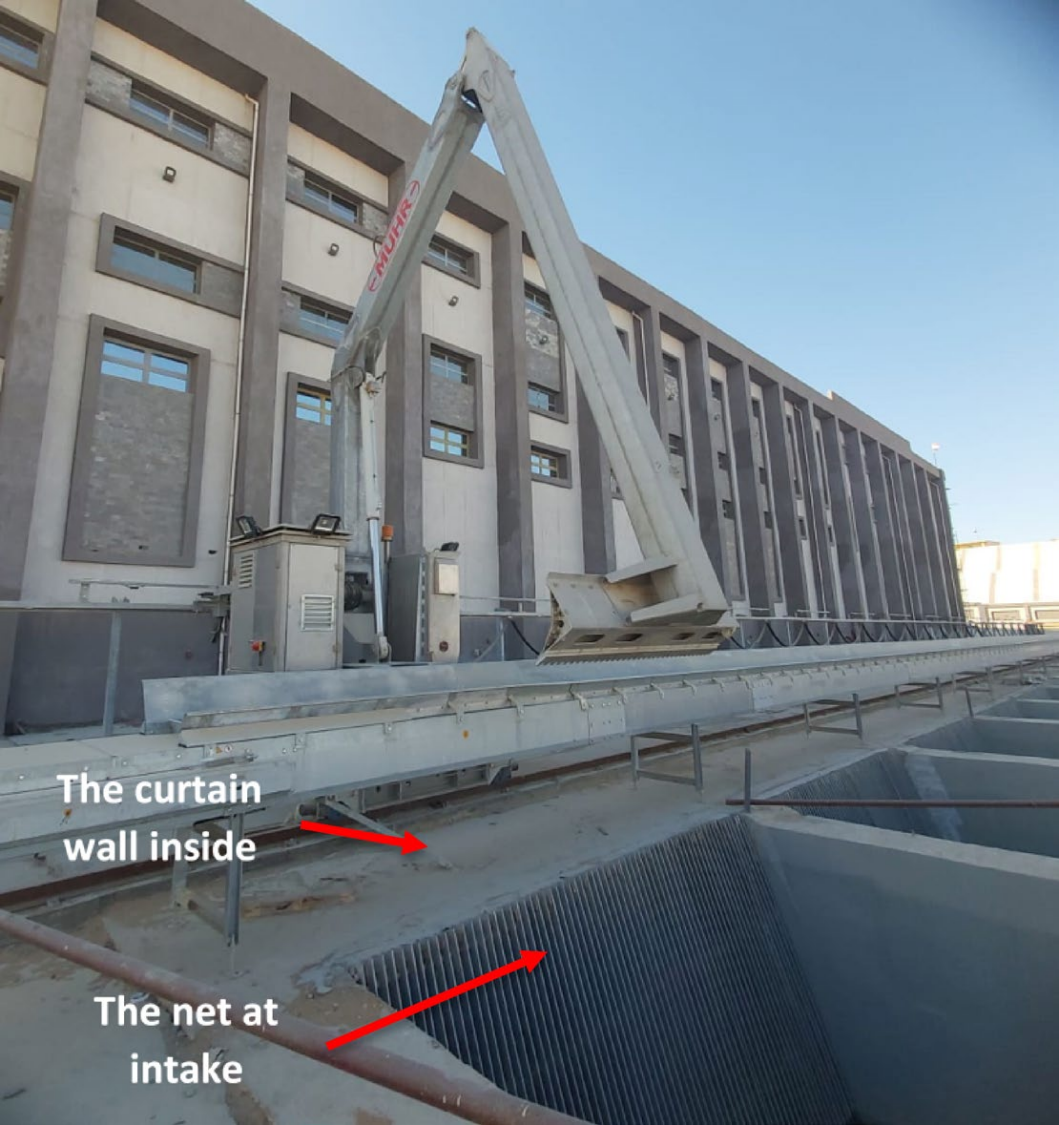
The curtain wall construction after pump intake nets.

**Fig. 17:**
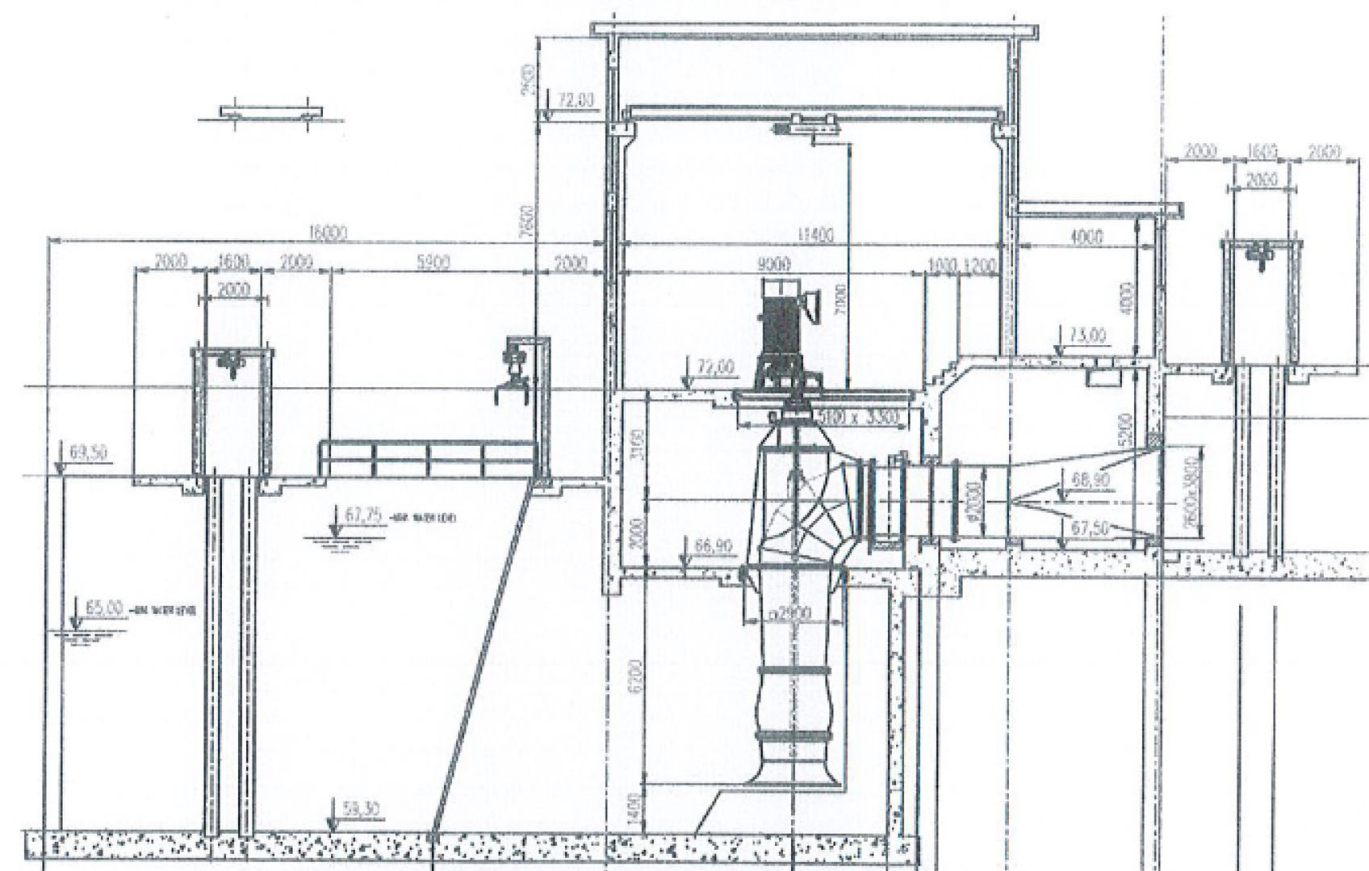
Scheme diagram for the intake of the pumping station.

The most important consideration in adopting a numerical simulation method is ensuring the accuracy of the calculation. To verify the effectiveness of the suggested modification to the pump intake by installation of the curtain wall to reduce the vortices. Inside the suction building, the curtain wall built after the pump intake nets as shown in Figs. 16 and 17 shows the scheme diagram of the pumping station. The proposed modification has been applied to the station intake as a trial to assess the effectiveness of the suggested change in reducing vortices. Moreover, all pump units are tested on a high-precision test to evaluate the vibration level after applying the pump intake modification.

**Fig. 18:**
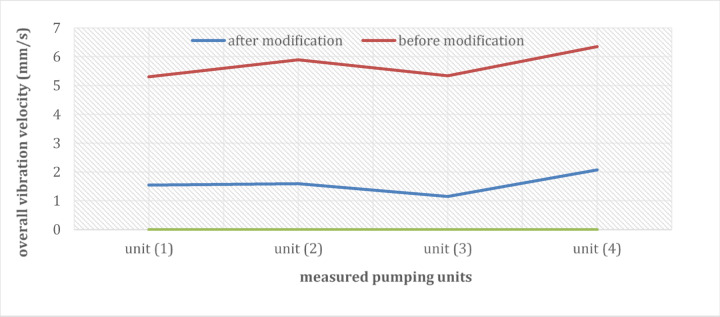
A comparison between overall vibration levels measured before and after applying modifications.

Dynamic analysis after execution of the new modification to the pump intake was repeated. The new measurements of the overall vibration level indicated a noticeable change as shown in Fig. 18. Measurements indicated that vibrations are extremely reduced. Maximum vibration level for pump unit (1) reached 1.54 mm/s on the motor non-drive end side which reduced by about 60% after the implementation of new intake modifications. For pump unit (2), maximum vibration level reached 1.59 mm/s on the motor non-drive end side which reduced by about 73%. Maximum vibration level reached 1.15 mm/s on the motor non-drive end side which reduced by about 65% at pump unit (3). For pump unit (4), maximum vibration level reached 2.07 mm/s on the motor non-drive end side which reduced by about 67%.

**Fig. 19:**
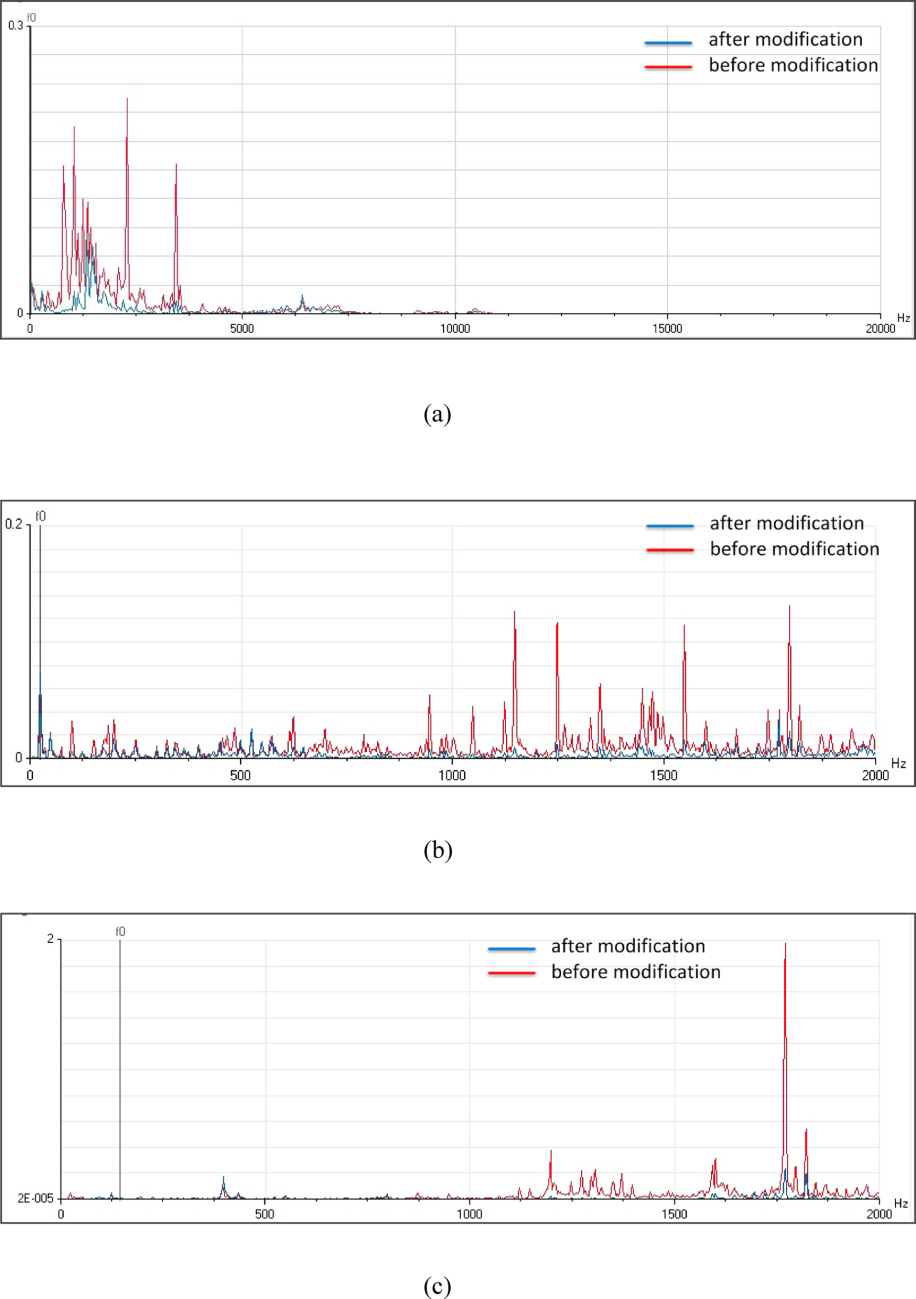

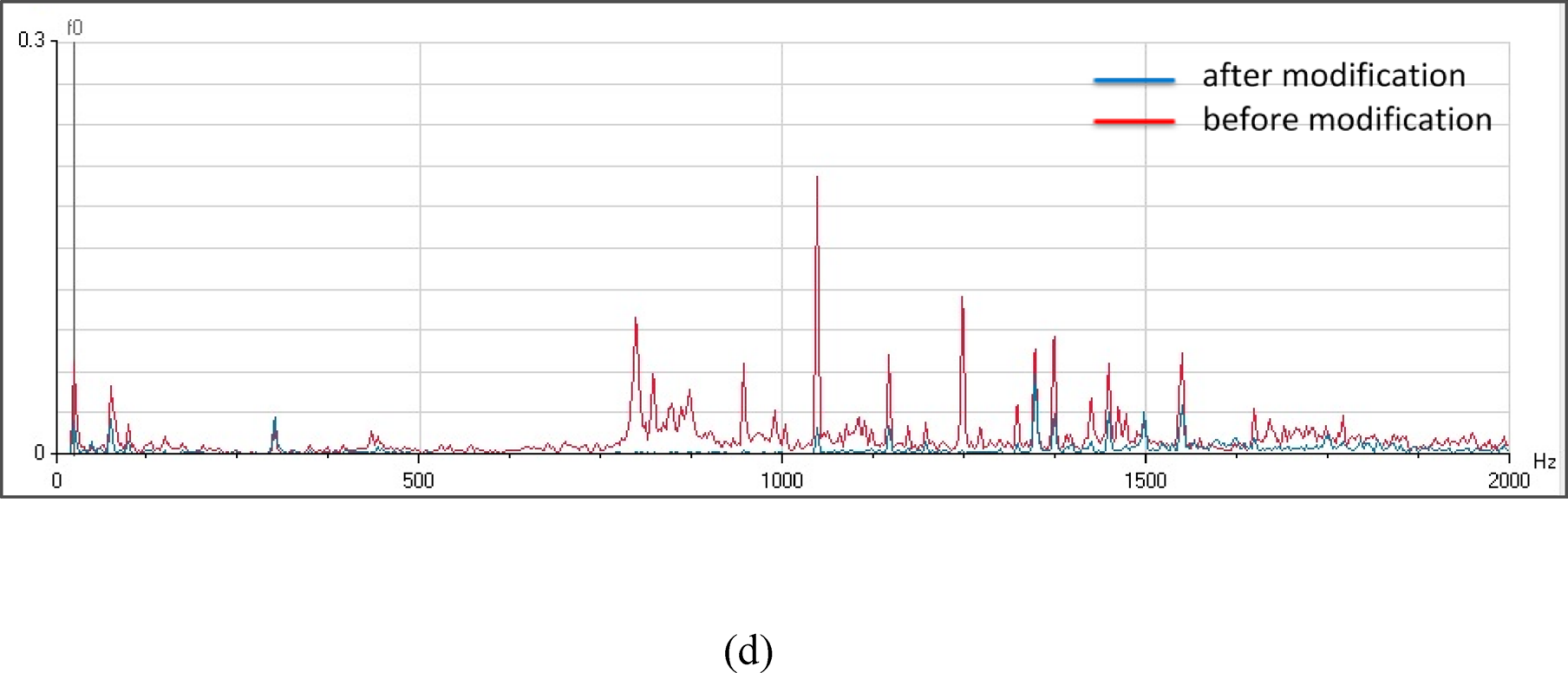
(**a**) Vibration spectrum of the pump unit (1) before and after modification. (**b**) Vibration spectrum of the pump unit (2) before and after modification. (**c**) Vibration spectrum of the pump unit (3) before and after modification. (**d**) Vibration spectrum of the pump unit (4) before and after modification.

The observed reduction in vibration amplitude for all pumping units following the applied modifications, as illustrated in Fig. 19, is indicative of a significant improvement in the system’s mechanical stability. The most significant of these factors are probably (1) a decrease in resonant frequencies because of the curtain wall’s construction and (2) a more uniform distribution of loads and forces within the system as a result of the lack of concentrated points of force brought about by vortices. These factors contribute to a more harmonious and efficient operation of the pumping units, potentially leading to increased reliability, reduced maintenance costs, and enhanced overall performance.

Pumps installed inside of pumping stations are impacted by the formation of vortices at the intakes. The overall vibration levels observed for all four pumping units show instability throughout operation hours. Frequency analysis of the station’s pumping units has been performed at multiple measurement locations to determine the excitation sources, since substantial problematic variations in overall vibration levels are usually caused by hydraulic difficulties.

Velocity spectrum analysis at the monitoring points exhibits evident fixed significant frequency equal to pump vane pass frequency (VPF) in axial, horizontal, and vertical directions. Minor peaks at the low frequency range also remain dominant. Acceleration spectrum analysis indicated notable increase in frequency amplitude within 1000- 10000Hz range that looks like a hump in the spectrum. Vane pass frequency is dominant for all measurement locations correlated to flow instabilities. Time wave form indicated many pulsations which are not evenly spaced. The results showed a hydraulic issue that has significant effects on the vibration signal. CFD analysis is used to apply a detailed understanding of the attributes of the sump flow. The results verified the vortex’s formation.

The vortex patterns at different intake points are explored to reveal the vortex shape change. The vortex disturbs the inlet flow fields of the impeller, resulting in significant reductions of the axial velocity weighted average angle and the axial velocity uniformity. The vortex increases the inlet passage hydraulic loss and affects the dynamic stability of the pump leading to high vibration levels. Because the emerge time of the vortex is not continuous, and the location of the vortex is not fixed the vibration levels are not stable with time. Analysis of the velocity uniformity shows low level of flow uniformity at the pump intake.

## Conclusion

This study investigated the primary cause of unstable and fluctuating vibration levels in the pumping station’s units. The research strategy involved combining the collection and analysis of vibration signals with a numerical simulation of the water intake of the pumping station. Key findings and conclusions are as follows:

1. Vibration magnitudes changed significantly close to the blade pass frequency when a vortex developed. Also, a vortex’s presence was successfully recognized at the measuring locations due to the abrupt increase in vibration strength.
2. The flow instability in the 1000–10,000 Hz frequency range have an impact on the vibration amplitude so, the frequency amplitude intensity increases significantly in this range.
3. The time series shows non-constant behavior in the frequency amplitude. Furthermore, changes in flow intensity are found to cause variations in the time–frequency characteristics.
4. The abrupt increase in vibration strength at the testing spots could be used to detect the presence and growth of vortices at the pumping unit’s input.
5. The CFD method may yield precise numerical findings about the vortex development along the pump intake, which is crucial for pump station intake design.
6. A curtain wall is a workable and appropriate solution to the vortex issue. Maximum vibration level reduced by about 73% after execution of the curtain wall to the pump intake.

This study demonstrates an innovative and effective solution to an actual problem in large pumping stations. The findings provide valuable guidance for future pump design, operation, and maintenance practices to ensure optimal performance and reliability.

## Data Availability

The data that support the findings of this study are available from the corresponding author upon reasonable request.
